# Dr. Joseph Curtis on Aconite Plaster in Rheumatism

**Published:** 1842-06-18

**Authors:** Joseph Curtis


*Aconite Plaster in Rheumatism.* By Joseph Curtis, M. D.—Since my communication appeared in the Lancet upon the external use of the tincture of aconite in cases of rheumatism, I have adopted a new mode of using it, namely, in the form of a plaster. The following is the way in which I make it:—

Take of the tincture of aconite ℥iv., evaporate to about ℥ss., or until it becomes of the consistence of oil. This should be spread with a paint brush upon one yard of adhesive plaster half a yard wide, and dried. This plaster may be cut to any convenient size and shape, and applied to the part affected.

The effect of this plaster is so nearly the same as that of the tincture applied as before described, that it will not be worth while occupying your columns with cases. When first put on, its effects are much milder than those of the tincture; in fact, it produces so comfortable a glow, that I seldom find a patient in any hurry to part with it. I believe all its beneficial effects are generally produced in less than twenty-four hours; if allowed to remain on three or four days it will sometimes bring out a rash.—*Lon. Lan.* April 2, 1842.

